# Implications of Oxytocin in Human Linguistic Cognition: From Genome to Phenome

**DOI:** 10.3389/fnins.2016.00271

**Published:** 2016-06-14

**Authors:** Constantina Theofanopoulou

**Affiliations:** Department of General Linguistics, University of BarcelonaBarcelona, Spain

**Keywords:** oxytocin, language, alpha rhythm, OXTR, POU3F2, LNPEP, FOXP2, CNTNAP2

## Abstract

The neurohormone oxytocin (OXT) has been found to mediate the regulation of complex socioemotional cognition in multiple ways both in humans and other animals. Recent studies have investigated the effects of OXT in different levels of analysis (from genetic to behavioral) chiefly targeting its impact on the social component and only indirectly indicating its implications in other components of our socio-interactive abilities. This article aims at shedding light onto how OXT might be modulating the multimodality that characterizes our higher-order linguistic abilities (vocal-auditory-attentional-memory-social systems). Based on evidence coming from genetic, EEG, fMRI, and behavioral studies, I attempt to establish the promises of this perspective with the goal of stressing the need for neuropeptide treatments to enter clinical practice.

## Introduction

The nine amino acid peptide oxytocin (OXT) is involved in an array of physiological and pathophysiological processes, with some of those most commonly reported in the literature being pregnancy and uterine contractions, milk ejection, sexual activity, pain modulation, social interaction and bonding, parental care, and attention to socially-relevant stimuli (for a good review see Meyer-Lindenberg et al., 2011). From another perspective, malfunctions of the oxytocinergic system have been reported in cases of Autism Spectrum Disorder, Schizophrenia, Obsessive Compulsive Disorder, Phobia, Prader-Willi Syndrome and Williams Syndrome, providing strong functional links to the social and emotional modules that all these cases share (Leckman et al., 1994; Lopatina et al., 2012; De Berardis et al., 2013; Grinevich et al., 2015; Haas and Smith, 2015). This broad perspective of the literature indicates that OXT impacts a wide spectrum of neurobehavioral systems.

**Figure 1:**
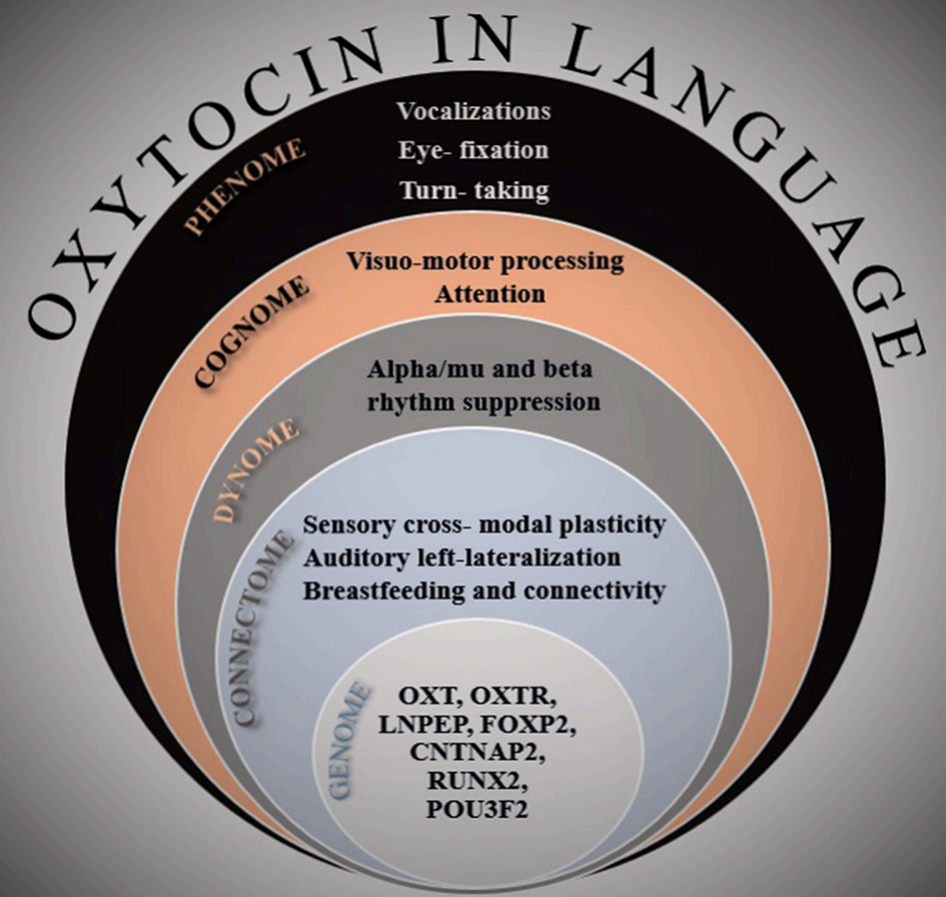
**A multi-dimensional illustration of the evidence presented in the paper**. At every level of analysis, the most important findings that are related to the role of oxytocin in linguistic cognition are listed.

Here I put forth the hypothesis that OXT also has a significant role in our linguistic abilities, ranging from modulating genes involved in spoken-language acquisition to modulating our motivation to communicate. In building this hypothesis, I follow an approach I have argued in Theofanopoulou and Boeckx (2015) in the context of cognitive phylogenies, where for a hypothesis to be valid in the Language Sciences, there needs to be evidence at multiple levels of biological organization, from genetics to ultimately the behavioral level (Fisher, 2015). Thus, I appeal to relevant findings from a multitude of studies, touching upon all the following levels of analysis: genome, connectome, dynome (brain oscillations), cognome, and phenome (See Figure 1). I also develop my hypothesis from a translational viewpoint among non-human animal studies and humans, including the molecular studies of OXT to its social functions in communication. I conclude that OXT most probably globally affects brain components that are tightly interwoven with the pinnacle of our social expressions, namely the sensory, motor, and more cognitive facets of our linguistic abilities (auditory, vocal, attention, and memory systems).

## Genome: OXT modulates genes involved in spoken-language acquisition

Apart from the aforementioned actions of OXT, what is of most relevance for the present article is its key role in several developmental processes that subserve the acquisition of our higher cognitive skills. Oxytocin-mediated, experience-dependent cross-modal plasticity in the sensory cortices during early development (Zheng et al., 2014) and the left-lateralized expression of OXT in the auditory cortex of the mouse brain (Marlin et al., 2015) suggest that OXT pathways are highly pertinent to understanding the sensory ontogeny of our linguistic communication. For humans, epigenetic misregulation of the OXTR via aberrant gene silencing with DNA methylation has implicated OXT in the development of Autism Spectrum Disorder, where deficits in language performance are included in its core phenotype (Gregory et al., 2009). A potential mechanism is that epigenetic DNA methylation of the oxytocin receptor gene (OXTR) is associated with neural activity and functional coupling of neurons (Puglia et al., 2015). Thus, the aberrant OXTR expression by methylation could be impacting neural activity and neuronal coupling in language performance.

An even more possible direct genetic link between OXT and our linguistic capacities is evidenced in the robust findings with genes known to be necessary for normal language development, namely in the FOXP2-CNTNAP2 pathway. To begin with, interaction between OXT and CNTNAP2 in critical developmental windows has been shown in a mouse model of autism (Peñagarikano et al., 2015). FOXP2 regulates CNTNAP2 expression, and CNTNAP2 has been linked to complex neurological disorders, including language impairment, autism, dyslexia, schizophrenia, and depression, with Single Nucleotide Polymorphisms (SNPs) having been associated with specific language endophenotypes (see Rodenas-Cuadrado et al., 2014 for review).

Another link between OXT and FOXP2 is provided through LNPEP, the peptidase that metabolizes oxytocin, located on chromosome 5q15 (for more details on LNPEP see Ebstein et al., 2012). Vernes et al. (2007) identified genomic sites directly bound by FOXP2 protein in native chromatin of human neuron-like cells, and LNPEP was among the genes with the most robust and consistent binding. LNEP functionally regulates synaptic transmission and formation.

A third potential interaction between OXT and FOXP2 may occur by two other genes related to language: (i) RUNX2 and (ii) POU3F2 (Benítez-Burraco and Boeckx, 2014, 2015). For RUNX2, –a critical transcription factor for osteoblast formation-, Tamma et al. (2009) found that it was differentially regulated in OXT knockout mice. RUNX2 is connected to many genes that are essential not only for brain and language development, but also for bone formation (Boeckx and Benítez-Burraco, 2015). A direct interaction between RUNX2 and FOXP2 has been experimentally demonstrated in the context of endochondral ossification (Zhao et al., 2015), a finding further reinforced by Gascoyne et al. (2015), who added FOXP2 to the list of established osteoblast and chondrocyte transcription factors (such as RUNX2). Significantly, the action of OXT on osteoblast maturation (Di Benedetto et al., 2014) and its implication in an osteogenic network that supports the development of our language-ready brain (and skull) may provide genetic evidence for the hypothesis that OXT may directly foster encephalization and our craniofacial phenotype (Carter, 2014). Last but not least, both OXT and RUNX2 have been found to be strongly connected to the Vitamin D endocrine system (Prüfer and Jirikowski, 1997; Han et al., 2013; Patrick and Ames, 2014), which has been proposed to explain the genetics and epidemiology of Autism (Cannell, 2008).

Concerning POU3F2, a transcription factor, neuronal and endocrine components (including OXT) of the hypothalamic-pituitary axis have been shown to be critically dependent on POU3F2 action (Nakai et al., 1995; Schonemann et al., 1995; Burbach et al., 2001). POU3F2 also regulates FOXP2 gene expression in a human-specific manner (Maricic et al., 2013). Crucially, the fact that in all three genes, OXTR, POU3F2, and FOXP2, there have been identified signs of positive selection in human or recent hominin evolution (Enard et al., 2002; Maricic et al., 2013; Schaschl et al., 2015), reinforces the idea that these evolutionary changes might be partially responsible for the emergence of aspects of our species-specific cognitive and linguistic abilities.

## Connectome

Recent studies have implicated OXT in brain development and plasticity. Specifically, the oxytocinergic brain system has been described to undergo major morphological alterations that modify the conformation of its neurons and glia and its synaptic inputs in a stimulus-dependent manner (Theodosis, 2002). The bulk of the evidence coming from studies in mice, rats and praire voles elucidates the significant role OXT plays in shaping different pathways of the brain (see Carter, 2003 for review). Importantly, the expression of the OXTR displays a particular maturational progression in the brain of the developing rat that could be classified in two types: transient expression during early postnatal development and constant abundant expression mediating neuronal transmission in the mature brain (Yoshimura et al., 1996). Similarly in mice, neocortical OXTR binding exhibits a transient peak in early postnatal periods, when extensive synaptic proliferation and pruning takes place (Hammock and Levitt, 2013).

These findings along with the ones that address the effect of the maturation of the OXT system on sensory—and not only socio-sexual- aspects could exemplify why early postnatal life is indeed a sensitive period for OXT in modeling circuits that are eventually responsible for sensory performance. Additional insight can be gained from comparative data on mice: Zheng et al. (2014) found that OXT promotes excitatory synaptic transmission in the sensory cortices at a much earlier stage than the hitherto understood functions of OXT in social and emotional contexts and, notably, Marlin et al. (2015) found that both OXT receptors and projections from hypothalamic OXT-producing neurons are present in the auditory cortex of mice, with the former being more numerous on the left side than on the right, something that could be telling for lateralization in human language development (Theofanopoulou, 2015 and references therein).

In humans, it has not yet been experimentally established how early adjustments of the OXT system influence the neuronal and synaptic substrates that underlie the sensory and cognitive modules of our language-ready brain. The only (rough) conclusions we can deduce from the literature are based on comparisons between infants that have or have not been breastfed and concomitant brain changes. On the grounds that OXT is stable in milk and that OXT in maternal blood can be transferred to milk and then to neonates (Takeda et al., 1986), we would expect that lactation goes hand-in-hand with proliferating brain connectivity. At least, some evidence suggests so: Deoni et al. (2013) showed an association between early exclusive breastfeeding with increased development in late maturing white matter regions (interestingly also near BA44, traditionally linked to language). Tellingly, breastfed children also showed improved receptive language scores compared to formula-fed children. Moreover, Khedr et al. (2004) found that visual evoked potential (FVEP), brainstem auditory evoked potential (BAEP), and somatosensory evoked potential (SSEP) are more mature in breastfed infants relative to formula-fed infants at 1-year of age, something suggestive of the importance of breastfeeding in early development. I propose that an important molecule and factor could be the high concentration in OXT in breast milk and also its release during skin-to-skin contact over breastfeeding (Uvnäs-Moberg et al., 2015). Furthermore, the aforementioned results (see also Kafouri et al., 2013 and Isaacs et al., 2010) mesh well with recent studies showing that autism is to a great extent correlated with inefficient breastfeeding, by cause of lack of interest in milk-suckling (Williams et al., 2000; Gallup and Hobbs, 2011; Al-Farsi et al., 2012; Steinman and Mankuta, 2013). A deeper understanding of the complex OXT feedback loop between mother and infant in breastfeeding could be reached if we additionally take into account that the perturbation of the system might be actually stemming from the mother. Indeed, birth complications (Brimacombe et al., 2007) due to low OXT levels and stressful-depressive mother care have long been associated with autism (see Uvnäs-Moberg et al., 2015 for an excellent review on the short- and long-term effects of breastfeeding and skin-to-skin contact between mother and infant, explained via OXT release). According to this thread of interpretation, traditional psychological theories on the role of the “refrigerator-mother” in the etiology of autism could now be construed on a neuroendocrine basis.

Another important issue at the level of the connectome is the loci where OXT is expressed in the brain. In humans, OXT is dispersed from the magnocellular neurons in the paraventricular and supraoptic nuclei of the hypothalamus to practically throughout the brain: including the amygdala, the hippocampus, the striatum, the brainstem, the cerebellum, the insula, the suprachiasmatic nucleus, the septum, the bed nucleus of stria terminalis, the globus pallidus, the substantia nigra pars compacta, the ventral tegmental are, the spinal cord, and to neocortical areas traditionally associated with “language,” such as the prefrontal cortex, the anterior cingulate cortex and the precuneus (Lee et al., 2009, 2010; Ma et al., 2016). Even though it is important to find out “where” OXT is expressed in the brain, a mere locationist approach cannot enlighten our understanding of “how” OXT gives rise to cognitive sub-processes mechanistically (Theofanopoulou and Boeckx, 2015). At the following level of analysis (i.e., the dynome) the direct effects of OXT administration on brain rhythms and how this translates into specific cognitive processes (i.e., the cognome) will be illustrated.

## Dynome—cognome

Only very recently attempts have been made to link the action of OXT with a rhythmic correlate in the human brain that would make some sense in terms of its cognitive significance. In early experimental attempts of pure behavioral paradigms (e.g., “trust” experiments, for example: Baumgartner et al., 2008), OXT was not implicated at a granularity level that could be matched with the (de)activation of a specific oscillatory band. It was not until 2009, when Kéri and Benedek examined the effect of OXT on the perception of biological vs. non-biological motion stimuli, that a venue for associating OXT modulation to neural activity was opened (Kéri and Benedek, 2009). Specifically, Kéri and Benedek found that OXT enhances the ability to detect biological motion in noise, whereas no such effect turned up when detecting a rotating shape. This led Perry et al. (2010) to tentatively link these results with the alpha/mu and beta brain rhythms, which have been shown to be suppressed while observing actions executed by someone else (Muthukumaraswamy and Johnson, 2004; Lange et al., 2015). Characteristically, alpha/mu and beta rhythms have been found to be desynchronized reinforcing the efficiency of the mirror neuron system, which in humans is activated not only when observing biological actions, but also at all levels of communicative interactions (see Pineda, 2005 for a review). This is more than pertinent to the scope of this article, since for linguistic communication interplay to happen, it is necessary not only to perceive biological movements (lip-movements, tongue-movements, formant transitions, hand gestures, and eye movements), but to couple them with the auditory input and whence make out the multidimensional meaning of the compound “linguistic” input. As I have put forward elsewhere (Theofanopoulou, 2015), this interplay should be mediated by an attentional mechanism that keeps track of all these distinct rhythmic stimuli. It should not take us by surprise then that an overall decrease of the aforesaid rhythms has also been linked to increased demands of attention and memory (Klimesch, 2012). Importantly, after OXT administration, alpha/mu and beta rhythms had a general suppressive effect that was widespread across the scalp (viz not only on brain areas of the somato-motor cortex), something that was interpreted as an effect on a broader network, in which mirror/motor and attentional mechanisms can be with difficultly disentangled (Perry et al., 2010). A similar experiment was conducted by Singh et al. (2015), also in Schizophrenia patients, and replicated the diffused effect of OXT in the brain. Lastly, Hepker (2016) tested how OXT affects mirror neuron activity in a hand-gesture experiment and encountered greater mu rhythm suppression, in accordance with other experiments, but this time for a biological movement directly involved in language processing.

To the best of my knowledge, there are no studies yet showing that OXT has a direct effect on the rhythmic patterns in a purely linguistic task. But as put forth in Theofanopoulou (2016) there are several reasons to expect so. Firstly, alpha/mu and beta band suppression have been shown to coordinate the rhythms partaking not only in motor but also in auditory (speech) (Obleser and Weisz, 2012) processing and OXT seems to support this multimodality, considering that it has been found to increase not only in response to biological motions, but also to vocalizations alone (Seltzer et al., 2010) and to attenuate the human acoustic startle response (Ellenbogen et al., 2014). Secondly, in autism alpha-band deployment was shown to be severely impaired, giving rise to increased distraction (Oberman et al., 2005, 2008; Murphy et al., 2014; see also Moran and Hong, 2011, for similar findings in schizophrenia). Here magnetoencephalography (MEG) studies showing atypical auditory responses in patients with autism are also of relevance: for example, in autistic patients stronger responses to nonspeech than speech sounds (Yau et al., 2015), delayed (Roberts et al., 2010), and atypically lateralized (Orekhova et al., 2012) neuromagnetic auditory field responses compared to controls were observed. These experiments in conjunction with the irregularities observed in the oxytocinergic system in autism make it plausible that OXT might in part modulate the brain rhythms in language-processing.

## Phenome

In behavioral experiments OXT has been engaged in a surfeit of different complex tasks that can be difficult to decompose for the aims of this article. Accordingly, only experiments that are informative for different facets of linguistic processing will be mentioned.

OXT has been loosely associated with “communicative” functions (Yamasue, 2013) that only recently have been broken down into processes that correspond to more specific linguistic processes. For instance, Seltzer et al. (2010) found that children under stress show increased OXT levels after hearing maternal vocalizations and Watanabe et al. (2014) showed that intranasal OXT administration to autism-patients affects their decisions about social information with conflicting verbal and non-verbal contents. Lastly, Ellenbogen et al. (2014) found that intranasal oxytocin attenuates the human acoustic startle response independent of emotional modulation.

However, most data come from studies involving OXT in eye-gaze enhancement suggesting its plausible role in interpersonal communication (Guastella et al., 2008) and in inferring the mental state of others (Domes et al., 2007). Gamer (2010) explains that OXT increased the proportion of fixation changes toward the eyes across all expressions, and did not directly affect the efficiency of processing emotional faces *per se*. In light of studies clarifying the importance of eye gaze in the modulation of speech and co-speech gesture (Holler et al., 2014, 2015), we can better appraise why in most cases the communicative deficits in autism derive from an abnormal fixation to the mouth region of the interlocutor, instead of the eye region (Pelphrey et al., 2005; Neumann et al., 2006). Tellingly, for therapeutic concerns, Andari et al. (2010) found that OXT selectively increased autism patients' gazing time on the eye region, improving their social performance.

In a similar vein, Ebitz and Platt (2014) further argue that these emitted eye-signals, regulated by OXT, provoke OXT-release back in the receiver, increasing eye contact and proximity seeking, establishing in this way a back-and-forward loop that strongly underlies communicative functions. This cascade of reciprocal OXT-secretion might, in other words, give a neurohormonal basis to the “turn-taking” roots of our linguistic capacity, recently highlighted from an evolutionary perspective (Levinson, 2016).

## Conclusion

In this article I attempted to draw attention to the potential implications of the neurohormone OXT in the context of language. Even though its role in purely linguistic matters has so far been overlooked, there is already a plethora of evidence strongly suggesting that a better understanding of its function could be rewarding. Results from experiments at different levels of analysis (from genetic to oscillatory and behavioral) suggest that OXT could fit well in the recently addressed hypotheses that underline the “reward-learning” foundations of our linguistic capacities (see Berra, 2015 for a good review). However, till now only dopamine has been tested in linguistic tasks in humans (Ripollés et al., 2014) and widely in vocal-learning in zebra finches (reviewed in Simonyan et al., 2012). More genetic experiments on the effect of OXT on mice vocalizations and birdsongs in different paradigms (courtship, affiliative, fear, dam-puppies) and EEG studies on its impact on alpha/mu/beta rhythm suppression in a speech perception task would help to appreciate more the role of OXT in our high cognition and its possible therapeutic implications.

## Author contributions

The author confirms being the sole contributor of this work and approved it for publication.

## Funding

Preparation of this work was supported by funds from the Spanish Ministry of Economy and Competitiveness (grant FFI2013-43823-P), as well as funds from the Generalitat de Catalunya (FI-grant-2016-2019).

### Conflict of interest statement

The author declares that the research was conducted in the absence of any commercial or financial relationships that could be construed as a potential conflict of interest.
